# Rheumatoid factor and anti-citrullinated protein IgA antibodies in the diagnosis, prognosis and monitoring of patients with rheumatoid arthritis

**DOI:** 10.1016/j.jtauto.2025.100332

**Published:** 2025-11-12

**Authors:** Ulrike Steffen, Daniela Sieghart, Günter Steiner

**Affiliations:** a Department of Medicine 3 - Rheumatology and Immunology, Friedrich-Alexander-Universität Erlangen-Nürnberg and Uniklinikum Erlangen, Erlangen, Germany; b Deutsches Zentrum für Immuntherapie (DZI), Friedrich-Alexander-Universität Erlangen-Nürnberg and Uniklinikum Erlangen, Erlangen, Germany; c FAU Profile Center Immunomedicine (FAU I-MED), Friedrich-Alexander-Universität (FAU) Erlangen-Nürnberg, Erlangen, Germany; d Division of Rheumatology, Department of Medicine III, Medical University of Vienna, Vienna, Austria; e Ludwig Boltzmann Institute for Arthritis and Rehabilitation, Vienna, Austria

**Keywords:** Rheumatoid arthritis, Rheumatoid factor, *Anti*-citrullinated peptide antibodies, Diagnosis, Prognosis, Extra-articular manifestations, Mucosal origin hypothesis

## Abstract

Over the past decades immunoglobulin A (IgA) antibodies have gained increasing attention for their diagnostic and prognostic significance in rheumatoid arthritis (RA), complementing the well-established roles of IgM rheumatoid factor (RF) and IgG anti-citrullinated protein antibodies (ACPA). IgA-RF and IgA-ACPA are found in a subset of RA patients and have been associated with more severe disease phenotypes, including increased joint erosion and extra-articular manifestations, especially cardiovascular disease and lung involvement. Moreover, concerning prediction of therapeutic responses IgA isotypes seem to have potential, as their presence has been shown to be associated with a blunted response to treatment with TNF inhibitors suggesting their usefulness for disease monitoring during follow-up once a diagnosis has been established. Although the diagnostic value of IgA autoantibodies in identifying seronegative RA cases is limited, their presence confirms a diagnosis of RA and may be helpful in the preclinical detection of individuals at risk for developing RA.

Increasing evidence suggests that IgA-RF and IgA-ACPA may contribute to disease pathogenesis. They can activate myeloid cells through engagement with the Fc alpha receptor I, leading to enhanced pro-inflammatory cytokine release, phagocytosis, and the formation of neutrophil extracellular traps that exacerbate tissue damage. Taken together, measuring IgA isotypes may be considered a valuable addition to the serological armamentarium for RA, with potential to improve early diagnosis, risk stratification, and personalized therapeutic approaches.

This review summarizes the current knowledge regarding the value of IgA-RF and IgA-ACPA as diagnostic and prognostic markers for RA. In addition, we discuss how the presence of IgA autoantibodies fits into the mucosal origin theory and describe their potential pathologic effects.

## Highlights

• IgA isotypes of RF and ACPA are specific disease markers of RA.
• IgA-RF is associated with more severe disease and worse outcome.
• IgA-RF is associated with extra-articular manifestations.
• IgA-RF and IgA-ACPA are associated with a blunted response to TNF inhibitor treatment.
• IgA istotypes of RF and ACPA may substantially contribute to the pathogenesis of RA.

## 1 Introduction

Rheumatoid arthritis (RA) is a chronic autoimmune disease marked by systemic inflammation and joint destruction. It is the most common and most severe inflammatory joint disease with a worldwide prevalence of about 1 % in adults, mainly affecting women [1,2]. RA is characterized by chronic joint inflammation leading to cartilage and bone damage which may result in disability, reduction of quality of life and increased mortality. Therefore serological markers are pivotal in RA diagnosis, prognostication and treatment guidance [3,4]. Herein autoantibodies play a central role since RA is a systemic autoimmune disease that is serologically characterized by the presence of autoantibodies in blood and synovial fluid [[5], [6], [7]]. Among these, rheumatoid factor (RF) and anti-citrullinated peptide antibodies (ACPA) have proven extremely useful for diagnosis and also have some prognostic value regarding disease progression and clinial outcome [8,9].

RF was the first RA-associated autoantibody identified [10]. RF is an antibody or rather a family of antibodies directed against the Fc part of immunoglobulin (Ig) G. This part of the molecule is essential for complement fixation and interaction with Fc receptors and thus for the uptake of immune complexes. Classically, and in contrast to most other autoantibodies of systemic autommune diseases, RF primarily occurs as IgM in 60–70 % of RA patients and less frequently as IgA or IgG. However, IgM-RF is only moderately specific for RA and may be present with lower prevalence (except for primary Sjogren's disease) also in other rheumatic diseases as well as during infections as part of the humoral immune response. However, chronic persistence of high-titer RF is a pathological feature of RA and assumed to play a pivotal role in the pathogenesis of this disorder [11].

ACPA are directed to epitopes containing the non-proteinogenic amino acid citrulline that is generated by posttranslational deiminiation of arginine by enzymes of the peptidyl arginine deiminase (PAD) family [6,12,13]. The major ACPA isotype is IgG which is detectable at disease onset in 50–60 % of RA patients and in <5 % of inflammatory disease controls. Thus, IgG-ACPA is considerably more specific than IgM-RF. The majority of ACPA positive RA patients also display RF and the combined presence of ACPA and RF has shown the highest disease specificity for RA and is rarely observed in other inflammatory joint diseases [14,15]. However, around disease onset RF/ACPA double positivity is detected in only about 50 % RA patients and therefore the diagnostic sensitivity is considered moderate. RF/ACPA positive patients are clinically distinct from seronegative patients showing a more severe disease course and extra-articular manifestations that are less frequently observed in seronegative patients. Interestingly, bone damage and disease activity seem to be more associated with RF than ACPA [11,16,17].

Among other isotypes of RF and ACPA, IgA antibodies are of particular interest because they are suspected to play a role in the pathophysiology of RA due to their association with disease severity and their potential mucosal origin [18]. IgA isotypes of RF and ACPA are detectable in serum, saliva and joint fluid [19,20] although the amounts in peripheral blood are much lower than IgM-RF and IgG-ACPA. Furthermore, there is some evidence supporting the usefulness of the IgA isotypes for diagnostic and prognostic application, but this is still a topic of debate [[21], [22], [23], [24], [25], [26]]. This review summarizes the current understanding of IgA-RF and IgA-ACPA in RA, discussing their immunological characteristics, diagnostic performance, association with disease activity and progression, and potential clinical applications.

## 2 Structure and function of IgA antibodies

**Fig. 1:**
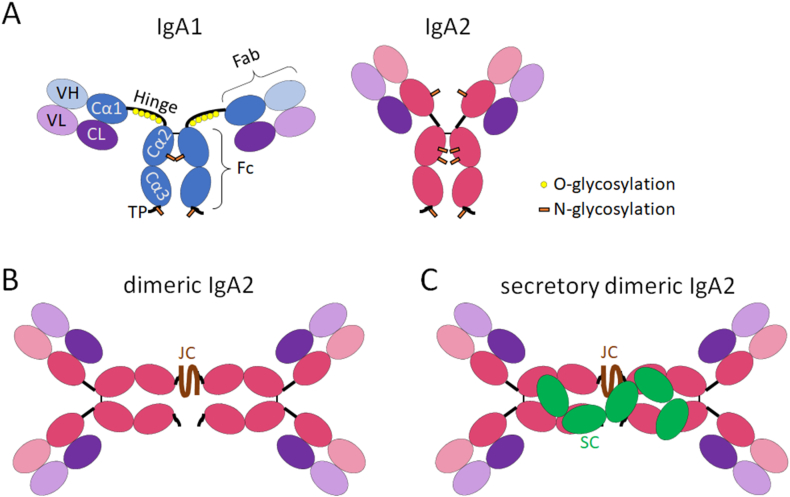
Schematic summary of the structure of (A) monomeric IgA, (B) dimeric IgA and (C) secretory dimeric IgA. Cα1-3 = constant domain 1-3 of the heavy IgA chain; CL = constant domain of the light chain; Fab = antigen binding fragment; Fc = crystallizable fragment; JC = Joining chain; SC = secretory component; TP = tailpiece; VH = variable domain of heavy chain; VL = variable domain of light chain.

Humans possess five antibody classes, or isotypes: IgA, IgD, IgG, IgE, and IgM. Like all antibodies, IgA is composed of two light chains (each comprising one variable and one conserved domain) and two heavy chains (each comprising one variable and three conserved domains) that are connected with each other via disulfide bridges (Fig. 1A). The light chains, together with the variable domain and the first conserved domain of the heavy chain, form the antigen-binding fragment (Fab), while the other conserved domains of the heavy chains form the crystallizable fragment (Fc part) (reviewed in Ref. [27]). IgA is the only antibody isotype that can be present in monomeric as well as in dimeric or, to a much lesser extent, other multimeric (trimeric, tetrameric, or pentameric) forms [28,29]. IgA dimerization is mediated by the formation of disulfide bridges between the tailpieces of two IgA molecules and the joining (J) chain (Fig. 1B).

Humans possess two IgA subclasses: IgA1 and IgA2, which differ mainly in the lengths of their hinge region and the number of glycosylation sites (Fig. 1A). IgA1 is the predominant form in blood [30]. The elongated hinge region of IgA1, which contains six O-glycosylation sites, distinguishes it from all other isotypes and has the effect that IgA1 has a T-shape rather than the classical Y-form of other isotypes, increasing the range and flexibility of its Fab regions [31]. IgA2 has a shorter hinge region that lacks O-glycans. Both IgA subclasses contain two conserved N-glycosylation sites within the Fc part and the tailpiece. IgA2 harbors two (in some allotypes 3) additional N-glycosylation sites compared to IgA1 [32]. In addition, the glycan composition varies between IgA1 and IgA2, with IgA1 generally exhibiting stronger processing, including higher degrees of galactosylation and sialylation. Of note, IgA1 and IgA2 appear to differ in their effector functions with IgA2 eliciting more NET formation and inflammatory cytokine production in neutrophils and CD103^+^ dendritic cells, putatively via the engagement of additional receptors [30,33].

With an estimated production rate of 50–130 mg/kg/day [34], IgA is the most abundantly produced antibody in the human body. Monomeric IgA remains in the blood, while dimeric IgA is rapidly secreted into mucosal areas. This secretion is mediated by binding of the dimeric IgA to the poly Ig receptor (pIgR) on epithelial cells, followed by transcytosis through the cells and release into the mucosa [35]. During this process, part of the pIgR is cleaved and remains bound to the IgA dimer, forming the so-called secretory component (Fig. 1C).

In mucosal areas, IgA plays an important role in first line defense and microbial homeostasis. IgA binds to microbes via its Fab region in an antigen specific manner and via interactions of the glycans on the IgA hinge region, the J chain, and the secretory component in an antigen-unspecific manner. Binding of IgA to microbes impedes their motility and possibly leads to the aggregation of neighboring cells, thereby preventing pathogens from approaching the gastrointestinal wall and infecting the host [36]. On the other hand, IgA appears to promote the maintenance of certain microbes in the gut, thereby shaping the microbiota by mechanisms that are still not fully understood [28]. In general, secretory IgA is considered anti-inflammatory. It was long thought that, in contrast to blood IgA, secretory IgA could not bind to its main effector receptor, the Fcα receptor I (FcαRI or CD89), due to steric hindrance via the secretory component. However, recent cryogenic electron microscopy data revealed that binding of secretory IgA to FcαRI is, at least in vitro, possible [28,37]. Whether the engagement of FcαRI by secretory IgA in complex with bound antigen occurs in vivo remains unclear.

Monomeric IgA which represents the most abundant form of IgA in the circulation, reportedly binds to the FcαRI and thereby activates immune cells [38]. FcαRI is primarily expressed on myeloid cells, including neutrophils, monocytes, macrophages, eosinophils and certain dendritic cells [[39], [40], [41]]. Like most activating FcRs, FcαRI has low affinity for monomeric IgA and needs to be crosslinked by complexed IgA. FcαRI signaling is mediated by the immunoreceptor tyrosine-based activation motif (ITAM)-harboring Fc receptor gamma chain (FcRγ) [42]. Activation of FcαRI results in various effector functions, including neutrophil extracellular trap formation, degranulation, phagocytosis, and antibody-dependent cellular cytotoxicity (ADCC) [43]. Interestingly, anti-inflammatory effects have also been described, which are mediated via inhibitory ITAM (ITAMi) signaling [44]. These anti-inflammatory effects appear to be primarily caused by transient binding of soluble monomeric or dimeric IgA, while complexed IgA elicits a full-blown response. Of note, FcαRI does not exist in mice, which makes in vivo functional studies difficult.

## 3 Diagnostic relevance of RF and ACPA IgA isotypes

### 3.1 IgA-RF

**Fig. 2:**
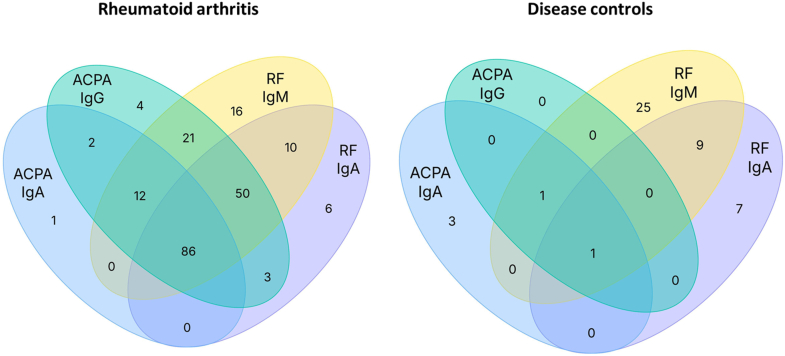
Overlap of IgA-RF and IgA-ACPA with IgM-RF and IgG-ACPA. Antibodies were measured in 305 RA patients and 263 disease controls including patients with osteoarthritis, reactive arthritis, axial spondyloarthropathy, osteoporosis, myositis, SLE, and ANCA-associated vasculitis [24]. As can be depicted from the Venn diagrams both IgA isotypes largely overlap with the routinely measured antibodies (IgM-RF and IgG-ACPA). With the notable exception of IgG-ACPA, none of the autoantibody isotypes is specific for RA when occurring in the absence of other antibodies.

From the early 80ies on an increasing interest has emerged in measuring IgA-RF in addition to the IgM isotype [45]. Several studies reported the presence of IgA-RF in approximately 50 % of RA patients compared to 60–70 % for IgM-RF and consistently showed higher specificity and positive likelihood ratios (PLR) of IgA-RF determined by ELISA as compared to the IgM isotype determined by nephelometry (largely measuring IgM) or IgM specific ELISA. A recently published meta-analysis reported IgA-RF specificity of 91 % (95 % CI 90.8–92.0 %) and a positive likelihood ratio of 7.7 [22]. Thus, IgA-RF has the highest diagnostic specificity among RF isotypes which is further increased in case of IgM-IgA double positivity [24,46,47] as depicted in Table 1 and in Fig. 2. This comes at the cost of lower sensitivity, but measurement of both RF isotypes might be helpful for the diagnosis of ACPA negative patients. However, it must be emphasized that single IgA-RF positivity (in the absence of IgG-ACPA and IgM-RF) is rarely seen in RA and not specific (Fig. 2).

**Table 1:** Diagnostic performance of IgA-RF and IgA-ACPA as compared to IgM-RF and IgG-ACPA. Antibodies were determined in 305 RA patients and 263 disease controls [24]. For details refer to legend of Fig. 2. IgA-ACPA was nearly as specific as IgG-ACPA but considerably less sensitive. IgG-ACPA showed highest specificity and PLR (73.0) followed by IgA-ACPA (17.4), IgA-RF (7.7) and IgM-RF (4.4). Combined presence of IgA-RF and IgM-RF increased specificity of RF testing substantially (PLR 12.6). Remarkably, specificity and PLR of IgA-RF + IgA-ACPA double positivity was comparable to IgG-ACPA. Of note, in this cohort of patients with established diagnoses, the specificity of IgG-ACPA was higher than in most other studies or in cohorts of early arthritis patients [15,51,52].

	IgA-RF	IgM-RF	IgA + IgM RF	IgA-ACPA	IgG-ACPA	IgA + IgG ACPA	IgA RF + ACPA
**Sensitivity**	49.8 %	60.0 %	47.9 %	33.1 %	58.4 %	32.8 %	28.2 %
**Specificity**	93.5 %	86.3 %	96.2 %	98.1 %	99.2 %	99.2 %	99.6 %
**PLR**	7.7	4.4	12.6	17.4	73.0	41.0	70.5

PLR - Positive likelihood ratio.

### 3.2 IgA-ACPA

IgA-class ACPA show very high specificity for RA that is comparable to that of IgG-ACPA, ranging from 95 to 98 % in early and established RA (Table 1). However, IgA-ACPA are considerably less prevalent than the IgG isotype or IgA-RF [21,24,48]. Thus, IgA-ACPA positivity ranges from 20 to 40 % of patients as compared to 50–60 % of patients who are IgG-ACPA positive. Critically, the vast majority of IgA-ACPA positive individuals test also positive for IgG-ACPA. Thus, due to the almost complete overlap measuring IgA-ACPA rarely increases diagnostic sensitivity but adds confirmation rather than identifying new seropositive cases. In a study recently performed with early untreated RA patients, an assay combining IgG and IgA ACPA showed slight gains in sensitivity but at the cost of lower specificity unless the cutoff was stringently adjusted. In detail, the CCP3.1 third-generation test detecting both isotypes was slightly more sensitive than the IgG CCP2 second generation assay but showed only moderate specificity of 91 %; when the cutoff was raised, specificity and sensitivity were comparable to the CCP2 assay measuring IgG only [23].

### 3.3 Presence of IgA isotypes in other diseases

Despite the high disease specificity of the IgA isotypes in the context of rheumatic diseases, it must be emphasized that IgA-RF can occur in other chronic inflammatory states (cirrhosis, chronic infections), and IgA-ACPA have also been found in patients with idiopathic pulmonary fibrosis where the IgA isotype was more prevalent than IgG-ACPA [49]. A similar observation was made in patients with inflammatory bowel disease [50]. In sharp contrast to RA, in inflammatory bowel disease neither IgM- nor IgA-RF was elevated suggesting a mucosal origin of IgA-ACPA in this disease which is less clear in RA (see section 5). Therefore, isolated IgA-RF or IgA-ACPA positivity should be cautiously interpreted in clinical context.

### 3.4 Overlap of IgA isotypes with IgM-RF and IgG-ACPA

The IgA isotypes of RF and ACPA largely overlap with the respective autoantibodies in their predominating isoform and patients positive for IgA-RF or IgA-ACPA are frequently also positive for IgM-RF and/or IgG-ACPA Therefore the added diagnostic value of IgA determination is rather limited (Fig. 2). Although additional measurement of IgA-isotypes may slightly increase sensitivity of RF and ACPA determination, it needs to be emphasized that RA is characterized by the presence of multiple specificities and that single reactivities are not sufficiently specific for RA [14,24]. IgA-ACPA or IgA-RF in the absence of other antibodies may be found in similar (low) frequency also in disease controls (Fig. 2) and therefore their isolated appearance needs to be interpreted with caution, particularly when clinical symptoms are unspecific. However, the presence of IgA isotypes further supports a diagnosis of RA in clinically suspect patients positive for IgG-ACPA and/or IgM-RF. Remarkably, combined presence of IgA-RF and IgA-ACPA is almost exclusively observed in RA and as specific as IgG-ACPA (Table 1). Of note, sera positive for the two IgA isotypes are often also positive for IgG-ACPA and IgM-RF.

## 4 Relevance of RF and ACPA IgA isotypes for disease prognosis and monitoring

### 4.1 Disease progression, severity and outcome

#### 4.1.1 IgA-RF

While the added diagnostic value of IgA class autoantibodies is limited, both IgA-RF and IgA-ACPA seem to have a considerable prognostic value since they are linked to more aggressive RA phenotypes. As early as in 1984, it was noted that IgA-RF portends erosive disease, given that IgA-RF levels correlated with bone erosions and fluctuations in disease activity [53,54]. Subsequently, IgA-RF was shown to be associated with disease progression, worse outcome and extra-articular manifestations, such as interstitial lung disease and cardiovascular complications whereas such associations were not found or much less pronounced for IgM-RF. Thus, patients with IgA-RF tend to have higher disease activity, more joint damage, and more extra-articular manifestations compared to RF-negative patients [[53], [54], [55], [56], [57], [58]]. The prognostic value of IgA-RF in early RA was underscored in a prospective study, showing that IgA-RF positivity at disease onset was an independent predictor of radiographic progression over the next several years. Even when controlling for IgG-ACPA status, baseline IgA-RF was associated with greater joint damage accrual [59]. In line with these data, IgA-RF was found to be among the earliest antibodies in pre-symptomatic individuals antedating onset of symptoms by several years [60]. Remarkably, in samples collected more than 15 years before disease onset, the IgA-RF isotype was significantly more prevalent than the other RF or ACPA isotypes [61]. Together, these findings suggest that the previously observed association of RF seropositivity with severe RA may be largely driven by the IgA isotype. However, data have been inconsistent across studies. A systematic review collating 36 studies found conflicting results on RF isotypes and outcomes: roughly half the studies linked IgA-RF to more severe radiological progression, while others did not find significant associations. Although the presence of multiple RF isotypes did correlate with higher likelihood of RA and possibly worse outcomes, no single isotype consistently predicted prognosis across all studies. This inconsistency may stem from assay variation (discussed in section 4.5), varying definitions of “poor outcome” (erosions, disability, etc.), different time spans, and treatment differences between studies [22].

#### 4.1.2 IgA-ACPA

Since in most of the earlier investigations the presence of ACPA was not yet considered, IgA-ACPA have been examined more recently as a marker of aggressive disease. Because IgG-ACPA itself strongly predict erosive progression it is challenging to parse the independent effect of IgA-ACPA. In the first study addressing this issue it was found that the occurrence of IgA-class antibodies was associated with smoking, and IgA-ACPA positive early RA patients had a more severe disease course over 3 years compared with IgA-ACPA negative cases, portending pathogenetic implications and a potential prognostic value of IgA-ACPA [26]. A long-term observation study revealed that IgA-ACPA baseline levels predict radiographic progression after 11 years of treatment [62]. Although IgA-RF was not included in the analysis it is reasonable to assume that a similar predictive value would have been found for this antibody given the high overlap of IgA-RF and IgA-ACPA.

#### 4.1.3 Multiple isotypes

Some studies have proposed that the presence of ACPA in multiple isotypes (IgG and IgA, or even IgM) denotes a more mature or robust humoral autoimmune response that could accelerate pathology [14,24]. The assumption is that an expanded isotype response (class-switching to IgA, etc.) might indicate a longer-standing or more intense immune drive, portending worse outcomes. Thus, RA patients with multiple ACPA isotypes, including both IgM- and IgA-ACPA, had more severe radiographic progression than those lacking the IgA isotype, although high IgG-ACPA titers remained the dominant predictor of damage [63]. Furthermore, the presence of multiple ACPA isotypes before disease onset was shown to be highly predictive of later development of erosive RA [64]. Similar observations were made for RF isotypes, bolstering the prognostic value of multiple isotype testing [60,61]. However, some early RA studies did not find a clear additive prognostic value for IgA-ACPA, once IgG-ACPA was accounted for. This was illustrated in a treat-to-target cohort which specifically evaluated IgA-RF and IgA-ACPA in early arthritis patients. In this investigation, IgA-RF and IgA-ACPA showed no significant independent association with 3-year outcomes (including sustained remission, DMARD-free remission, or need for biologics) after accounting for the conventional IgM-RF/IgG-ACPA status. Since almost all IgA-positive patients were positive for the standard isotypes the small excess risk seen in univariate analysis disappeared on multivariate analysis [21].

### 4.2 Extra-articular manifestations

Apart from radiographic progression, significant association of IgA-RF with extra-articular manifestations was demonstrated in a study published already three decades ago revealing that 80 % of IgA-RF positive patients had one or more extra-articular RA complications, versus only approximately 20 % of IgA-RF negative patients with elevated IgM/IgG-RF who were not different from seronegative patients [56]. In line with this early report, IgA-RF has been repeatedly found associated with rheumatoid pulmonary involvement [[65], [66], [67]] and a recent study suggested IgA-RF to be a potential predicting factor for poor prognosis of RA patients suffering from interstitial lung disease [68]. A diagnostic value has been attributed to IgA-RF also for assessment of rheumatoid vasculitis which together with decreased levels of complement C3 increased the probability of histologically proven rheumatoid vasculitis [69]. Moreover, in a recent study addressing association of IgG-ACPA and RF isotypes with cardiovascular events in patients with RA IgM-RF as well as IgG-ACPA were associated with future cardiovascular events including acute coronary syndrome, stroke and major adverse cardiovascular events. Remarkably however, only IgA-RF was exclusively associated with cardiovascular related death [70].

In summary, IgA-RF (and to a lesser extent IgA-ACPA) has been associated with higher disease activity, more erosions, and extra-articular disease in many studies. IgA-RF at baseline can predict radiographic damage progression, and the combination of IgA isotypes with other autoantibodies signals a more robust autoimmune response. However, since most IgA positive patients show high levels of the standard autoantibodies IgM-RF and IgG-ACPA the necessity of IgA antibody testing for outcome prediction remains questionable. The prognostic utility of IgA antibodies may thus rather lie in identifying patients at risk for extra-articular manifestations or lung disease rather than general prognosis, and more prospective studies are needed to better validate the prognostic power of IgA isotypes.

### 4.3 Therapeutic decision-making

One area in which IgA antibodies are showing promise is predicting how patients will respond to specific treatments. Emerging evidence suggests that seropositivity – and IgA isotypes in particular – may influence how patients respond to biologic therapies. Notably, the clinical response to tumor necrosis factor inhibitors (TNFi) has been linked to RF status, particularly to IgA-RF. A study published in 2007 showed that RA patients who failed to respond to TNFi were found to have significantly higher IgA-RF levels than responders, and in multivariate analysis IgA-RF was the only autoantibody independently predicting a blunted response [71]. This result was subsequently confirmed by two smaller studies [72,73]. Interestingly, patients with high levels of all RF subtypes (IgM, IgA, IgG) failing on TNFi therapy responded adequately to B cell depletion therapy and the difference between responders and non-responders was more significant for IgA-RF than for IgM-RF [72].

**Fig. 3:**
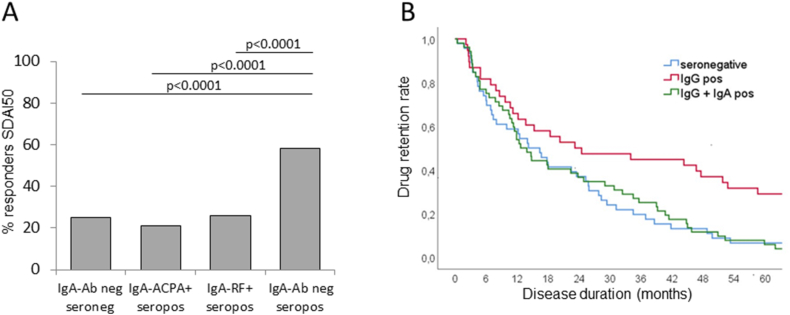
Association of IgA-ACPA with a diminished response to TNF inhibitory therapy. (A) Almost 60 % of seropositive patients testing negative for IgA isotypes (n = 58) showed a SDAI50 response (i.e. a 50 % reduction in SDAI) while the percentage of responsive patients was significantly diminished in the largely overlapping subgroups of IgA-ACPA positive (n = 29) or IgA-RF (n = 33) positive patients which did not differ from the seronegative group (n = 19). (B) The drug retention rate was significantly lower in IgA-ACPA/IgG-ACPA double positive patients and not different from seronegative patients as compared to IgG-ACPA positive patients without IgA-ACPA [23].

In line with these findings, also the IgA-ACPA status seems to have similar prognostic implications for therapy (Fig. 3). IgA-ACPA positive RA patients showed significantly diminished responses to TNFi as measured by the simplified disease activity index 50 (SDAI50) indicating 50 % improvement of symptoms [74]. A similar result was obtained when the American College of Rheumatology 20 (ACR20) response criteria were applied (not shown). This was bolstered by a significantly diminished retention rate of TNFi [23]. Remarkably, retention rates of IgG-ACPA seropositive patients were significantly reduced when IgA-ACPA was additionally detectable and comparable to those of seronegative patients. Of note, the majority of the IgA-ACPA positive patients were not only positive for IgG-ACPA but also for both RF isotypes indicating strong immune activation (see Fig. 2). In contrast, only a subset of the IgA-ACPA negative patients showed multiple reactivities. Thus, IgA-ACPA rather than IgA-RF could potentially identify a subset of patients with a highly activated immune system less likely to respond to TNF inhibition, steering treatment toward alternative modes of action such as inhibiting signal tranduction pathways or targeting the adaptive immune system.

Beyond TNF inhibitors, it is less clear how IgA autoantibodies affect response to other treatments. Patients highly seropositive for RF and/or ACPA (who are often positive also for IgA isotypes) respond better than seronegative patients to B-cell depletion (rituximab) and T cell inhibition (abatacept), likely because their disease is more driven by autoimmune processes [[75], [76], [77]]. Nevertheless, it must be noted that biological therapies targeting the adaptive immune system may also be effective in the seronegative population.

Taken together, while the IgA isotypes are associated with a blunted response to TNFi high levels of IgM-RF and IgG-ACPA are associated with a more favourable response to B cell depletion and T cell inhibition irrespectively of the presence of IgA antibodies. This would argue to include determination of IgA subtypes into routine diagnostics but more prospective studies are needed to justify inclusion of the IgA status in treatment guidelines or recommendations.

### 4.4 Disease monitoring and clinical implications

Another consideration is whether IgA autoantibodies could be used to monitor disease course or remission given that biomarkers for monitoring disease course are still scarce [4]. Generally, IgA- and IgM-RF levels fall with effective therapy whereas IgG- and IgA-ACPA levels remain more or less constant. Furthermore, IgA-RF seems to be slightly more associated with disease activity and severity than IgM-RF. In one study, multiple regression analysis revealed IgA-RF to be the unique variable independently associated to severe joint damage [65], and in patients with early RA both IgA-RF and IgM-RF were associated with inflammation, in contrast to IgG-RF and IgG-ACPA [17]. In another study addressing changes in ACPA isotype levels in relation to treatment response, the most pronounced changes were observed for serum levels of secretory IgA-ACPA which decreased rapidly among patients with a good response to treatment whereas total IgA-ACPA levels remained more or less constant [78]. However, all these association are not strong enough to allow use RF levels as reliable indicators of treatment success. Moreover, since changes in IgA-RF often parallel IgM-RF changes they have so far not proven superior in tracking disease activity. Nevertheless, IgA isotypes seem to have some potential for disease monitoring, especially secretory IgA-ACPA, and more studies are needed to fully appreciate the value of IgA isotypes for this purpose.

One clear clinical implication of IgA autoantibodies is in the context of extra-articular RA as pointed out in section 4.2. If a patient has high IgA-RF, the clinician should be vigilant for extra-articular manifestations including lung involvement, cardiovascular complications or vasculitis given the known associations. For example, in an RA patient with pulmonary symptoms, a positive IgA-RF might reinforce suspicion of RA-associated interstitial lung disease. Thus, IgA antibodies and particularly IgA-RF may be serving as prognostic factors aiding in the identification of difficult-to-treat RA [79]. Conversely, in patients with apparent RA who lack IgM-RF/IgG-ACPA, the combined presence of IgA-RF and IgA-ACPA can confirm the diagnosis and might prompt screening for mucosal processes (such as chronic lung inflammation) that could be driving an IgA response.

### 4.5 Limitations

Despite their potential usefulness, IgA-RF and IgA-ACPA testing has limitations that curtail widespread use. Assay standardization is an issue because an international reference serum is available only for IgM/nephelometric RF and more recently also for IgG-ACPA [80]. Moreover, different laboratories use different cutoff values and methods, which can yield disparate IgM-RF results [47]. Cost and availability are practical concerns; adding IgA isotype tests increases expense and complexity which could be minimized by restricting IgA determination to baseline samples and/or during follow-up, particularly when a shift to anti-TNF blockade is intended in patients showing an unsatisfactory response to DMARD treatment. Furthermore, the strong correlation of IgA positivity with IgM-RF/IgG-ACPA positivity means their incremental clinical value is limited in many cases given that sera highly positive for the routinely measured antibodies will often contain IgA antibodies in addition. On the other hand, the prognostic value of IgA isotypes is evident and particularly their association with extra-articular manifestations may help to identify patients at risk in the earliest stages since IgA antibodies like other isotypes may be detectable several years before disease onset [60,61,64,81]. In addition, in individuals at-risk for developing RA they may be better predictors for progression to RA than other isotypes. Interestingly, in a recent study a predictive value was seen only for IgA1-ACPA but not for the IgA2 subtype which would certainly deserve further investigation [25].

## 5 Involvement of the mucosa and IgA autoantibodies in the pathogenesis of rheumatoid arthritis

### 5.1 Mucosal origin hypothesis

RA is a multifaceted disease with several genetic and environmental risk factors. Many environmental risk factors (such as smoking or periodontitis) affect mucosal tissue. Smoking as well as occupational inhalable agents (e.g. silica dust, textile dust, and asbestos) have been shown to strongly increase the risk for ACPA positive RA in people carrying HLA-shared epitope alleles [82,83]. It was thought that smoking induces citrullination of lung tissue and thereby contributes to the loss of tolerance [84]. However, another study showed that citrullinated proteins in lung tissue are found at similar amounts in smokers and non-smokers [85], suggesting that the relationship between smoking and RA is more complicated.

Whether periodontitis increases the risk to develop RA is being debated as studies generated inconsistent results. A recent meta-analysis showed an increased risk for periodontitis patients to develop RA [86]. The periodontitis-associated bacterium *Porphyromonas gingivalis* expresses a bacterial isoform of PAD distinct from the mammalian enzymes and could potentially contribute to initial anti-citrullinated immune responses [87]. However, a recent study showed that ACPA from RA patients don't bind to autocitrullinated *Porphyromonas gingivalis* proteins challenging this hypothesis [88].

Recently, the interplay of the gut and the immune system in the context of RA has received huge interest. Alterations in the gut microbiome, mucosal inflammation, and increased intestinal permeability have been described to occur not only in people diagnosed with RA, but even before disease onset [89,90], suggesting gut dysbiosis to play a role in RA onset and perpetuation.

Together, these findings led to the hypothesis of mucosal origin of RA. The presence of IgA-ACPA partly supports this hypothesis as IgA is the most frequent antibody isotype in the mucosa and thus mainly considered as a “mucosal antibody”. In several studies, smoking was associated with IgA-ACPA and IgA-RF positivity, whereas carriage of the shared epitope was associated with IgG-ACPA positivity [26,[91], [92], [93], [94]]. In ACPA, the mucosa-associated IgA2 subclass is overrepresented compared to total serum IgA [30] and increased fractions of polymeric IgA have been observed in IgA RF [95]. Moreover, ACPA have been found in various mucosal fluids of RA patients including saliva, sputum, and bronchoalveolar fluid [[96], [97], [98]], although they seem to be absent in the intestine [99]. Importantly, IgA isotypes of RF and ACPA are among the earliest detectable antibodies and may antedate disease onset by several years [60,64]. Remarkably, in one study, IgA-RF was the most prevalent isotype in serum samples collected >15 years before disease onset [61].

However, there are also some findings challenging mucosal origin of RA autoantibodies. The most notable finding is that IgA-ACPA are much less common than IgG-ACPA in RA patients. The majority of RA patients is IgG- and IgA-ACPA double positive or IgG-ACPA single positive, while IgA-ACPA single positive patients are rare [20,24,25,100]. If the initial breakdown of tolerance occurred in a mucosal area one would expect IgA-ACPA to be at least as prevalent as IgG-ACPA. In contrast to patients with RA, patients with the mucosal disease idiopathic pulmonary fibrosis display more often IgA-ACPA than IgG-ACPA [49]. In addition, investigation of periodontal inflammatory exudate revealed higher IgA-ACPA levels in healthy individuals than in RA patients [101]. Moreover, IgA-ACPA in serum or saliva of RA patients was not linked to periodontitis [102].

Taken together, whether the presence of IgA autoantibodies really reflects the involvement of antigen encounter in mucosal areas or simply indicates a stronger ongoing autoimmune response with elevated class switch processes still needs to be elucidated. Immunizing events in mucosal areas strongly favor the production of IgA, so the presence of high levels of IgA autoantibodies might indeed suggest an initial antigen encounter in the mucosa. However, in RA patients, autoimmunity is developing over years and especially ACPA display a tremendous number of mutations most likely obtained in multiple germinal center circles. As the IgA locus lies downstream of the IgG locus, and class switch from IgG to IgA is a frequent phenomenon, IgA-ACPA might be a marker of a continuously ongoing immune response. Indeed, sequencing of plasmablasts from individuals at-risk to develop RA revealed cross-isotype clonal families between the IgA and IgG plasmablast [103].

### 5.2 Potential pathogenic involvement of IgA-ACPA and IgA-RF

**Fig. 4:**
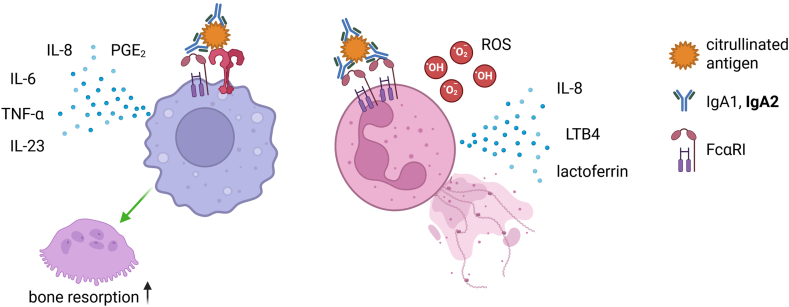
Potential pathogenic involvement of IgA-ACPA and IgA-RF. Immune complexes containing IgA-ACPA or IgA-RF bind to FcαRI on macrophages or neutrophils which leads to the release of cytokines, chemotactic factors, reactive oxygen species (ROS) and neutrophil extracellular traps. In addition, macrophages can promote osteoclast generation and bone resorption upon stimulation with IgA. IgA2 appears to have stronger inflammatory effects compared to IgA1. LTB4 = leukotriene B4; PGE_2_ = prostaglandin E2.

Although there is increasing evidence for the diagnostic and prognostic value of IgA-RF and IgA-ACPA it is still unclear whether or how much they contribute to the disease. However, some observations suggest that in addition to being a marker for a highly active immune response, IgA autoantibodies may contribute to the propagation of inflammation and potentially also to bone loss in RA (Fig. 4).

IgA can interact with neutrophils, monocytes, macrophages, and other myeloid cells via the IgA specific FcαRI. This interaction can elicit various effector functions, including phagocytosis, formation of neutrophil extracellular traps (NETs), antibody-dependent cellular cytotoxicity (ADCC), generation of reactive oxygen species (ROS), release of inflammatory cytokines, and antigen presentation [39,104,105]. Neutrophils, in particular, respond very strongly to IgA and show stronger responses to IgA than to IgG [106,107]. Importantly, neutrophils are one of the key players in RA. They are typically the first cells to enter the synovium in response to an inflammatory stimulus and propagate inflammation by attracting further immune cells through the release of inflammatory chemokines and cytokines. Upon degranulation, the release of ROS and proteolytic enzymes enhances tissue damage. Additionally, the release of NETs exposes citrullinated neoepitopes, which is believed to contribute to ACPA formation [108,109].

Markers of neutrophil activation and NET formation in the serum of RA patients have been described to correlate with disease severity and extra-articular manifestations, such as atherosclerosis and interstitial lung disease [[110], [111], [112], [113]]. Interestingly, NET markers are especially elevated in ACPA positive RA patients [114]. Autoantibodies can potentially trigger neutrophil activation and NET formation by engaging FcRs. Indeed, immune complexes isolated from the plasma and synovial fluid of RA patients resulted in the release of ROS, NETs, and chemotactic stimuli from isolated neutrophils [115]. Of note, this effect could be reduced by blocking FcαRI, suggesting that IgA-RF and IgA-ACPA contribute to inflammation through neutrophil activation. This hypothesis is supported by a study showing that in sputum of individuals at-risk to develop RA IgA-ACPA correlated with spontaneous NET formation of isolated neutrophils, as well as with NET remnant and inflammatory cytokine levels in sputum [116]. In addition to increasing NET formation, IgA has been shown to induce the release of the chemotactic factor leukotriene B4, which further enhances neutrophil infiltration [117].

In addition to neutrophils, macrophages express FcαRI and respond to IgA. IgA complexes alone often result only in a mild inflammatory response [30]. However, IgA and especially IgA2 seems to increase the inflammatory response of macrophages to toll-like receptor (TLR) stimuli [33,118]. Consistent with this observation, opsonization of bacteria with serum IgA stimulates the production of inflammatory cytokines, such as TNF-α, IL-1β, IL-6, and IL-23, in monocytes and macrophages [119]. Similar responses were observed with immune complexes of ACPA and RF isolated from the serum of RA patients [120]. This effect was inhibited by blocking antibodies against FcαRI, indicating that IgA-RF were the main drivers of inflammatory cytokine release. Recently it has been shown that ACPA of the IgA2 subclass increase the proinflammatory cytokine response of macrophages to TLR1/2 co-stimulation by eliciting metabolic changes [118], which may explain the observed association between IgA2:IgA1-ACPA ratios and RA disease severity and flares [30,100].

Besides a potential role in inflammation, there is some evidence that IgA autoantibodies might contribute to bone loss. Breedveld et al. showed that monocytes released IL-6 and IL-8 when stimulated with synovial fluid-derived immune complexes, and that the amount of the cytokines correlated with the levels of IgA-ACPA in the synovial fluid [121]. Culturing osteoclasts in the presence of supernatant from the IgA-activated monocytes, resulted in increased bone resorption. Supernatant of IgG-activated monocytes did not promote bone resorption as effectively, suggesting that IgA autoantibodies might contribute to bone loss by activating macrophages.

Together, these experimental results are consistent with the findings of several studies that RA patients who test positive for IgA autoantibodies develop more severe disease and experience more bone erosion than RA patients who test negative for IgA autoantibodies [55,122,123].

## 6 Concluding considerations

IgA-RF and IgA-ACPA have notable diagnostic and prognostic characteristics that have been elucidated over the past four decades. Both IgA isotypes are highly specific markers of RA but their clinical utility in diagnosis is limited by extensive overlap with standard serologies comprising IgM-RF and IgG-ACPA. Prognostically, abundant evidence links IgA-RF to more severe disease manifestations, including enhanced joint erosion and extra-articular complications, particularly interstitial lung disease. Furthermore, IgA autoantibodies might be helpful for predicting differential drug responses – particularly a reduced response to TNF inhibitors – but this insight has not yet been translated into formal treatment guidelines. Key limitations to a broader use of IgA-RF/ACPA include assay variability, low incremental yield over existing tests, and lack of prospective data demonstrating improved patient outcomes from their measurement.

Although the presence of IgA-RF and especially IgA-ACPA potentially supports the hypothesis of a mucosal origin of RA-related autoantibodies, there are also some facts speaking against this hypothesis. Of note, the presence of IgA-RF and IgA-ACPA associates with higher IgM-RF and IgG-ACPA titers. As a result of an ongoing immune response, B cells undergo class switch from IgM to IgG or IgA as well as from IgG to IgA. Thus, the presence of IgA autoantibodies might mainly reflect an increased autoimmune reaction (which likely enhances disease severity) rather than mucosal involvement. In addition, IgA autoantibodies can activate innate immune cells via FcαRI and potentially contribute to disease onset and propagation, although presumably to a lower degree than IgG-ACPA. In IgG-ACPA positive persons at-risk for developing RA, positivity for IgA-RF and/or IgA-ACPA (and thus for multiple isotypes and antibody specificities) associates with clinically suspect arthralgia and a highly increased risk to develop RA [25,[60], [61], [62],^124^,^125^]. The predictive value of IgA isotypes has to be validated in further at-risk populations but might indeed increase specificity of risk stratification criteria [126].

In conclusion, IgA-RF and IgA-ACPA serve as important biomarkers in research settings and may point to involvement of mucosal immune processes potentially driving RA. They confer high specificity for the disease and correlate with severe phenotype in many patients, on the one hand bolstering an RA diagnosis and on the other hand portending an aggressive course. Moreover, IgA-RF and IgA-ACPA may provide additional clinical information on therapy responses and clinical outcome. Therefore inclusion of IgA isotypes of RF and ACPA into routine diagnostics should be considered as they might be supportive for clinical decision making. Ongoing and future research will determine whether IgA antibodies can be leveraged for personalized RA care such as tailoring treatment choices or intensities in patients with high IgA-RF and/or IgA-ACPA. Until then, IgA isotypes may be viewed as complementary pieces of the complex puzzle of RA, enriching our understanding of disease heterogeneity and pathogenesis.

## CRediT authorship contribution statement

**Ulrike Steffen:** Writing – review & editing, Writing – original draft, Conceptualization. **Daniela Sieghart:** Writing – original draft, Investigation, Conceptualization. **Günter Steiner:** Writing – review & editing, Writing – original draft, Investigation, Conceptualization.

## Declaration of competing interest

The authors declare that they have no known competing financial interests or personal relationships that could have appeared to influence the work reported in this paper.

## Data Availability

No data was used for the research described in the article.
